# Unraveling the role of galectin-3 in cardiac pathology and physiology

**DOI:** 10.3389/fphys.2023.1304735

**Published:** 2023-12-18

**Authors:** Ignacio M. Seropian, Pablo Cassaglia, Verónica Miksztowicz, Germán E. González

**Affiliations:** ^1^ Laboratorio de Patología Cardiovascular Experimental e Hipertensión Arterial, Instituto de Investigaciones Biomédicas (UCA-CONICET), Facultad de Ciencias Médicas Universidad Católica Argentina, Buenos Aires, Argentina; ^2^ Servicio de Hemodinamia, Hospital Italiano de Buenos Aires, Buenos Aires, Argentina; ^3^ Departamento de Patología, Instituto de Salud Comunitaria, Universidad Nacional de Hurlingham, Buenos Aires, Argentina

**Keywords:** galectin 3, cardiovascular pathology, cardiac aging, cardiac remodeling, inflammation, myocardial infarction, atherosclerosis, healing

## Abstract

Galectin-3 (Gal-3) is a carbohydrate-binding protein with multiple functions. Gal-3 regulates cell growth, proliferation, and apoptosis by orchestrating cell-cell and cell-matrix interactions. It is implicated in the development and progression of cardiovascular disease, and its expression is increased in patients with heart failure. In atherosclerosis, Gal-3 promotes monocyte recruitment to the arterial wall boosting inflammation and atheroma. In acute myocardial infarction (AMI), the expression of Gal-3 increases in infarcted and remote zones from the beginning of AMI, and plays a critical role in macrophage infiltration, differentiation to M1 phenotype, inflammation and interstitial fibrosis through collagen synthesis. Genetic deficiency of Gal-3 delays wound healing, impairs cardiac remodeling and function after AMI. On the contrary, Gal-3 deficiency shows opposite results with improved remodeling and function in other cardiomyopathies and in hypertension. Pharmacologic inhibition with non-selective inhibitors is also protective in cardiac disease. Finally, we recently showed that Gal-3 participates in normal aging. However, genetic absence of Gal-3 in aged mice exacerbates pathological hypertrophy and increases fibrosis, as opposed to reduced fibrosis shown in cardiac disease. Despite some gaps in understanding its precise mechanisms of action, Gal-3 represents a potential therapeutic target for the treatment of cardiovascular diseases and the management of cardiac aging. In this review, we summarize the current knowledge regarding the role of Gal-3 in the pathophysiology of heart failure, atherosclerosis, hypertension, myocarditis, and ischemic heart disease. Furthermore, we describe the physiological role of Gal-3 in cardiac aging.

## 1 Introduction

Galectin-3 is a multifunctional carbohydrate-binding protein involved in a range of physiological and pathological processes. In recent years, there has been growing attention from both clinical and basic investigators on the role of Gal-3 in the regulatory mechanisms associated with cardiovascular physiology (Cassaglia et al., 2020; Fontana Estevez et al., 2022) and pathology (Funasaka et al., 2014; Gonzalez et al., 2014; de Oliveira et al., 2015; Cassaglia et al., 2020). It is now well-established that Gal-3 plays a pivotal role in the development and progression of cardiovascular diseases, including heart failure (de Boer et al., 2018), atherosclerosis (Pusuroglu et al., 2017), hypertension (Gonzalez et al., 2014), myocarditis (Souza et al., 2017a; Kovacevic et al., 2018), and ischemic heart disease (Wan et al., 2022; Wang et al., 2023). Furthermore, recent findings emphasize the significant contribution of Gal-3 to the temporal evolution of cardiac and renal aging, suggesting a key role in the mechanisms associated with organ senescence (Iacobini et al., 2005; Fontana Estevez et al., 2022).

Despite numerous investigations into the role of Gal-3 in cardiovascular physiology and pathology, many aspects of its specific mechanisms of action remain elusive. Nevertheless, given its crucial relevance to cardiovascular pathophysiology, Gal-3 emerges as a promising therapeutic target for the treatment of cardiovascular diseases. In this review, we aim to provide a comprehensive overview of the current knowledge regarding the role of Gal-3 in cardiovascular pathophysiology through its involvement in cardiac inflammation, fibrosis, and function, as well as its significance in cardiac physiology.

## 2 Galectin-3: synthesis, tissue distribution, and function

**FIGURE 1:**
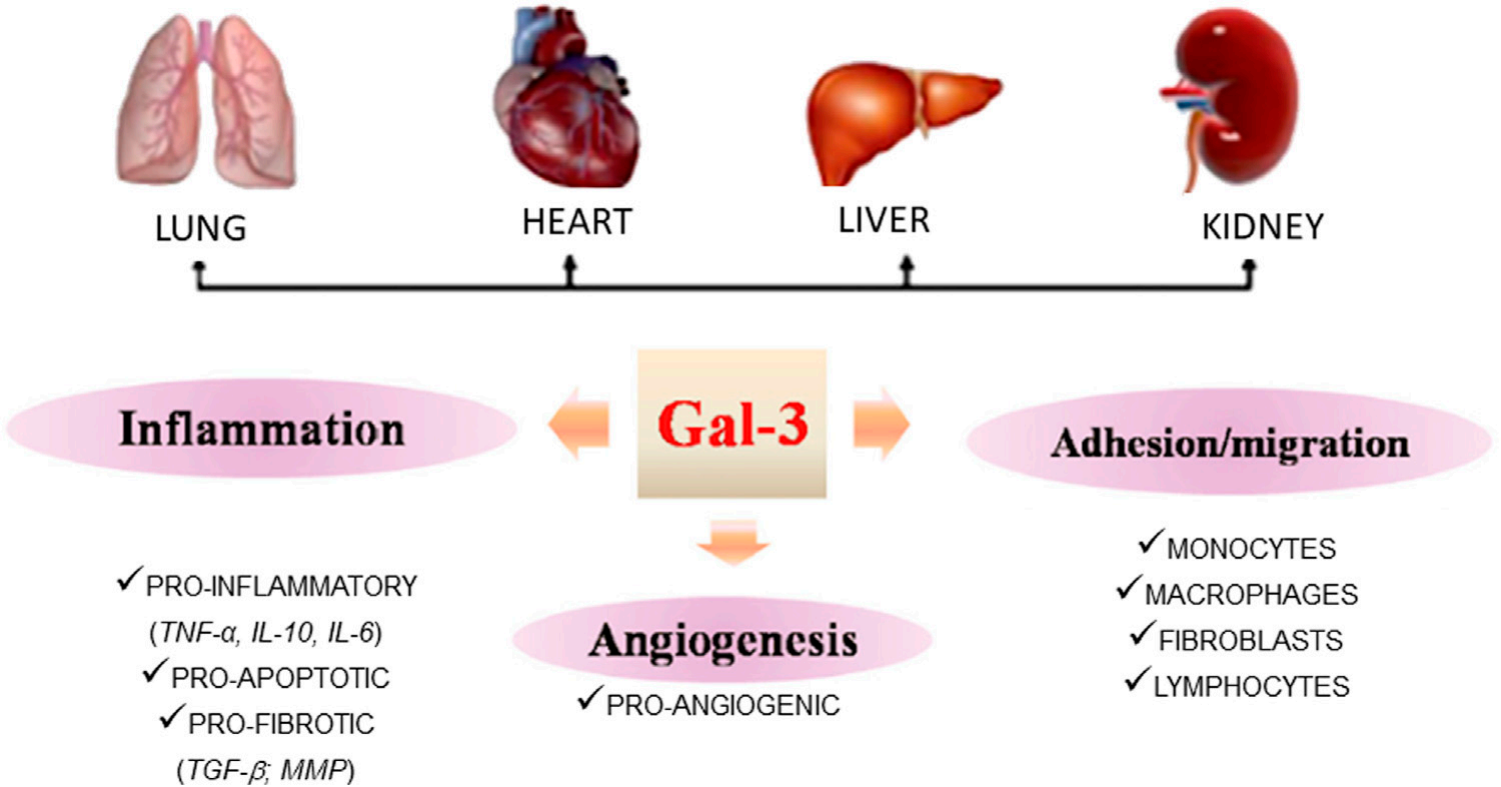
Representative picture of tissue expression of Gal-3 and its role on inflammation and angiogenesis.

Galectin-3, a member of the galectin family, is characterized by its unique ability to specifically bind to β-galactoside-containing glycans (Hovorkova et al., 2023). It stands apart from other galectins due to its chimeric nature, featuring a non-lectin domain that confers additional functional properties to the protein (Figure 1).

The constitutive form of Gal-3 is a monomer with an approximate molecular weight of 31 kDa. It comprises a conserved carbohydrate recognition domain (CRD) and a proline, glycine, and tyrosine-rich N-terminal non-lectin domain (NTD) (Diaz-Alvarez and Ortega, 2017). The CRD is responsible for Gal-3’s affinity for β-galactoside-containing glycans, while the NTD is pivotal in mediating protein-protein interactions, including binding to integrin and extracellular matrix (ECM) proteins (Dumic et al., 2006).

Gal-3 is primarily synthesized and localized within the cytoplasm and nucleus (Haudek et al., 2010). Galectins in general are soluble proteins synthesized on free ribosomes in the cytosol, and then rapidly secreted to the extracellular space through a yet unknown pathway (Johannes et al., 2018). At the extracellular level and on the cell surface, Gal-3 binds to plasma membranes and ECM glycoconjugates. It also engages in homo- and heterotypic interactions with other multivalent lectins. Intracellularly, Gal-3 plays a significant role in protecting against apoptosis, regulating cyclin D1 gene expression (Lin et al., 2002), and influencing alternative splicing. Extracellularly, Gal-3 acts as a chemotactic factor for various inflammatory cells and promotes the differentiation of endothelial cells and angiogenesis (Zhong et al., 2019), thus contributing to the development and progression of conditions such as tumors, neural degeneration, atherosclerosis, diabetes, and tissue repair (Funasaka et al., 2014). The multiple and varied roles of Gal-3 on different cells have consequences for several physiological processes related to immune responses and inflammation, as well as other pathological conditions such as fibrosis, cancer, and heart disease. Consequently, it is difficult to conclude if the final effect of Gal-3 on angiogenesis is beneficial or detrimental. While in certain pathological conditions such as myocardial hypertrophy, ischemia and reperfusion or even myocardial infarction the angiogenesis may contribute to enhance the tissue vascularization, in cancer it may contribute to tumor growth. Therefore, although the physiological role of Gal-3 on angiogenesis seems to be similar, the underlying pathology may lead to dissimilar consequence.

Gal-3 is widely expressed in cells from the lungs, spleen, stomach, colon, adrenal glands, uterus, ovaries, kidneys, heart, brain, pancreas, and liver (Yu et al., 2013; Nio-Kobayashi et al., 2014), particularly in inflammatory cells and endothelial cells (Leuschner et al., 2012; Funasaka et al., 2014; de Oliveira et al., 2015) (Figure 1). Notably, under pathological conditions; Gal-3 expression can be significantly upregulated, underscoring its crucial role even when constitutive expression is low, as is the case in the heart (Sciacchitano et al., 2018) (Table 1).

**TABLE 1:** Cardiac levels of Gal-3 in experimental models of cardiovascular disease.

Pathology	Model	Organ	Method	Result	Reference
AMI	Mice, Rats	Heart	mRNA, WB, RT-PCR RNA-Seq, ELISA	**↑↑↑**	Sanchez-Mas et al. (2014), Li et al. (2023), Mo et al. (2019), Zhang et al. (2020), Al-Salam and Hashmi (2018)
Hypertension	Mice, Rats	Heart, Kidney, VSMC, Blood	DNA array, WB, RT-PCR mRNA, cDNA, ELISA	**↑↑↑**	Kirk and de Boer (2019), Langheinrich et al. (1996), Frenay et al. (2015), Li et al. (2018), Lopez-Andres et al. (2011), Sharma et al. (2008), Lerman et al. (2019)
Atherosclerosis	Mice, Human	Plaque, Aorta	mRNA, WB, microarray	**↑↑↑**	Pang et al. (2023), Libby (2021)
Isoproterenol/Isoprenaline	Mice, Rat	Heart	mRNA, ELISA, WB, IHC	**↑↑↑**	Hu et al. (2023), Nguyen et al. (2018), Vergaro et al. (2016), Zhao et al. (2019)
Chagas Disease	Mice	Mesenchymal Stromal cells	mRNA	**↑↑↑**	Bern (2015)
Doxorubicin	Mice	Heart	ELISA, mRNA	**↑↑↑**	Souza et al. (2017b), Chatterjee et al. (2010), Zhang et al. (2021)
Myocarditis	Mice	Heart, Blood	ELISA, IHC, macrophages, mRNA, WB	**↑↑↑**	Zhang et al. (2011), Haudek et al. (2010)

AMI, acute myocardial infarction; WB, Western blot; RT-PCR, real time-PCR; ELISA, enzyme-linked immunosorbent assay; IHC, immunohistochemistry; VSMC, vascular smooth muscle cells.

Gal-3 play a pivotal role in a diverse array of biological events, exerting its influence on various cell types belonging to both innate and adaptive immunity. Its actions encompass leukocyte adhesion and migration through endothelial cells to reach sites of inflammation, recognition and clearance of microorganisms and structurally damaged cells, and the production of both pro- and anti-inflammatory cytokines in response to chemotactic agents (Pasmatzi et al., 2019). In the extracellular space, Gal-3 serves as a pattern recognition receptor (PRR) with the capacity to modulate the activity of innate immune cells, while also functioning as a damage-associated molecular pattern (DAMP) (Sato et al., 2009).

The binding of Gal-3 to glycans on cell surfaces and within the ECM elicits a wide range of effects on cellular behavior. Gal-3 binding can either promote or inhibit cell adhesion and migration, modulate the synthesis and activity of growth factors and cytokines, and regulate the expression of genes involved in inflammation, cell survival, and ECM remodeling (Ma et al., 2018).

The role of Gal-3 in inflammation is multifaceted and at times paradoxical. Most immune cells, including macrophages, neutrophils, and dendritic cells express Gal-3, affording it the capacity to regulate their activities through various pathways (Ma et al., 2018). Depending on the context and cell types involved, Gal-3 can function as a pro-inflammatory mediator either by enhancing immune cell recruitment and pro-inflammatory cytokine production, or as an anti-inflammatory agent by inhibiting immune cell activation and promoting apoptosis. Consequently, Gal-3’s exact role in inflammation is highly context-dependent. For instance, in the lungs, Gal-3 has been observed to promote inflammation resolution by inducing neutrophil apoptosis, whereas in the skin, it exacerbates inflammation by facilitating dendritic cell recruitment. Notably, recombinant Gal-3 induced neutrophil oxidative burst and endothelial cell adhesion through a process that depends on oligomerization (Almkvist et al., 2004; Sundqvist et al., 2013).

Inflammation is linked to fibrosis. Gal-3 stimulates collagen synthesis, thus participating in liver cirrhosis (Abu-Elsaad and Elkashef, 2016), pulmonary fibrosis (Jia et al., 2021), and cardiac fibrosis (Liu et al., 2009; Gonzalez et al., 2016). The mechanisms underpinning these effects involve the production of ECM proteins by fibroblasts, fibroblast differentiation into myofibroblasts, suppression of myofibroblast apoptosis, and release of pro-fibrotic cytokines by immune cells (Henderson et al., 2006; Jia et al., 2021). Experimental studies have demonstrated that the inhibition of Gal-3 ameliorates fibrosis in various animal models, suggesting that targeting Gal-3 may hold promise as an anti-fibrotic therapy (Henderson et al., 2006; Gonzalez et al., 2014; Chain et al., 2020; Jia et al., 2021).

## 3 Galectin-3 in cardiovascular disease

Gal-3 has emerged as a central player in the development and progression of heart failure and atherosclerosis. Increased expression of this chimeric lectin was observed in heart failure patients, and it is associated with heightened fibrosis and inflammation (Table 1). Gal-3 promotes heart failure through multiple mechanisms, including the infiltration of inflammatory cells, proliferation of fibroblasts, cardiomyocytes hypertrophy, and collagen synthesis. In this section, we will provide an overview of Gal-3’s role in myocardial infarction, hypertension, atherosclerosis, and other cardiomyopathies.

### 3.1 Myocardial infarction

Myocardial infarction is the main cause of heart failure. After the occlusion of a coronary artery, the ischemic cardiomyocytes undergo necrosis, and a healing process starts. This process is dynamic, with a sequence of structural and functional changes that includes the removal of necrotic cardiomyocytes to be replaced by a definitive fibrotic scar (Morales et al., 2002; Prabhu and Frangogiannis, 2016; Hanna et al., 2020). This scar should provide adequate tensile strength to prevent cardiac expansion, dilation, and rupture. Adverse remodeling is characterized by progressive global ventricular dilation and is associated with the temporal evolution of wound healing. While the mechanisms responsible for post-MI ventricular remodeling are multiple, varied, and complex, the magnitude of the inflammatory response during the repair of the infarcted area correlates to the progression of remodeling (Frangogiannis, 2014; Westman et al., 2016). Although an inflammatory response is necessary for the healing process, it needs to be balanced and controlled. An imbalance caused by excessive inflammation can lead to adverse ventricular remodeling and worse outcomes. On the other hand, inexistence of inflammation will impair the ability to produce a mature scar, with the increased risk of scar thinning and –ultimately- ventricular rupture. Shortly after AMI, necrotic myocytes release denatured macromolecules with antigenic function, activating signaling pathways associated with toll-like receptors, reactive oxygen species (ROS), and the complement system, which promote cytokine synthesis that stimulates the activation of the innate immune system leading to myocardial infiltration by polymorphonuclear leukocytes (PMN), and MMP activation. Subsequently, splenic monocytes are released and migrate to the infarct area where they infiltrate, accumulate, and participate in the repair process (Yap et al., 2023). Toll-like receptors (TLR) are pattern recognition receptors that ultimately promote inflammation through MYD88, IRAK and NF-kB. However, an MYD88 independent effect has also been described. Many cellular debris from necrotic cardiomyocytes bind and activate TLR-4 (Yang et al., 2016), and TLR-4 is the most studied receptor in AMI. Its inhibition is associated with improved cardiac remodeling and function (Prabhu and Frangogiannis, 2016). Gal-3 directly binds to TLR-4 resulting in TLR-4 activations and inflammation (Burguillos et al., 2015). However, these effects were studied in the brain, not in the heart. The complement cascade is also activated by necrotic cardiomyocytes in AMI and contributes to inflammation and reperfusion injury (Frangogiannis et al., 2002; Vogel, 2020). Although several mechanisms can activate the cascade, one of the final components is C5 activation leading to the generation of C5a is a very potent chemoattractant for neutrophils. *In vitro*, Gal-3 is necessary for glycosylated Ig1 to inhibit the chemoattractant capacity of C5a (Heyl et al., 2016). Moreover, Gal-3 directly inhibits PMN chemotaxis by C5a *in vitro* (Baseras et al., 2012). This effect is opposed to other pro inflammatory effects of Gal-3 in PMN, and Gal-3 can increase PMN chemotaxis *in vivo*, probably through mechanisms that do not involve C5a (Alves et al., 2013). ROS also contribute to inflammation and cardiac injury, especially injury after reperfusion (Frangogiannis et al., 2002). In experimental pulmonary arterial hypertension, Gal-3 promoted ROS, while Gal-3 inhibition decreased those (Fulton et al., 2019). PMN from Gal-3 KO mice also produced less ROS than WT mice after Toxoplasmosis infection (Alves et al., 2013). These effects are mediated by its ability to bind carbohydrates (Forsman et al., 2008).

**FIGURE 2:**
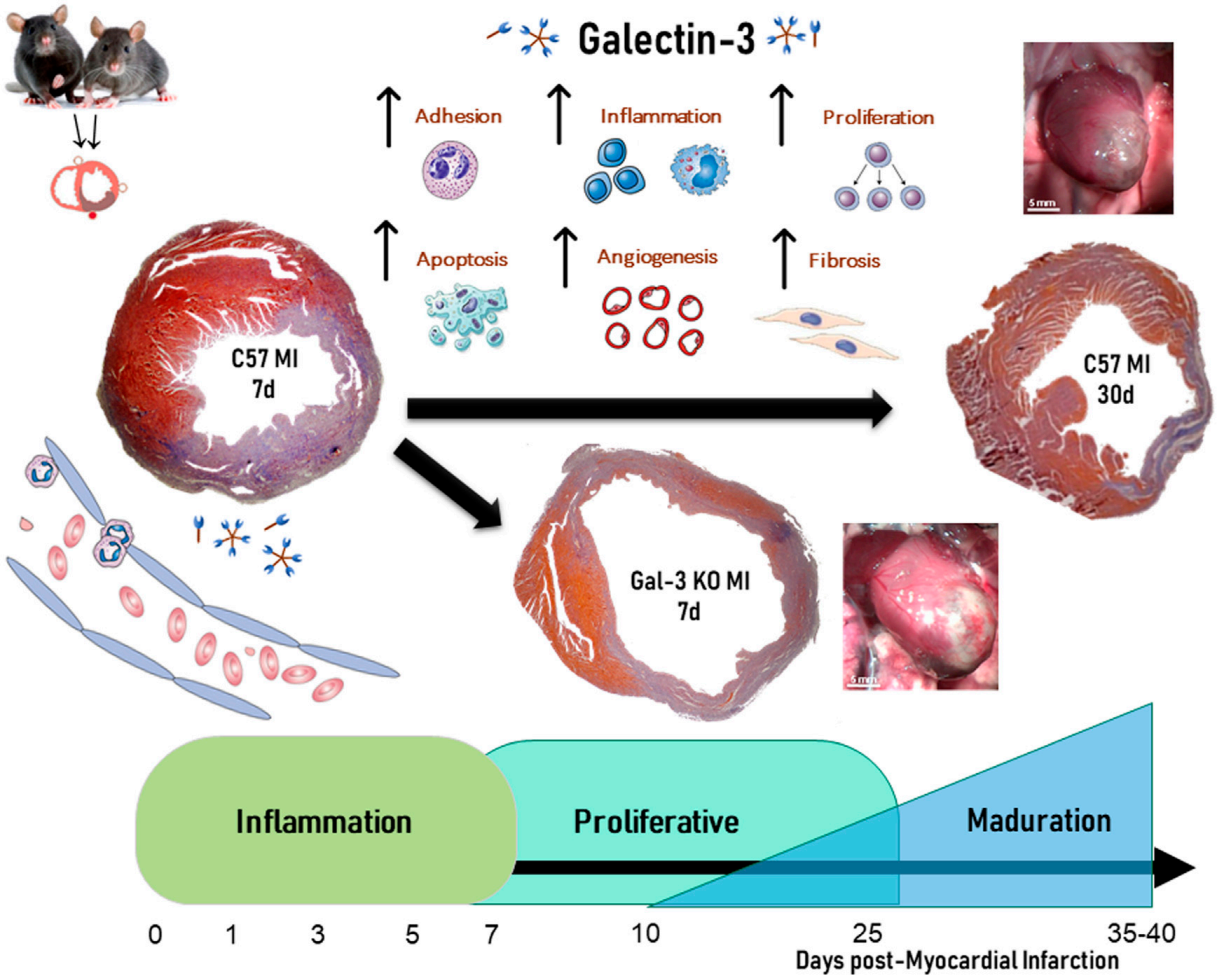
Role of Galectin-3 on temporal evolution of myocardial infarction.

Classically, the reparative process after AMI has been divided into three overlapping phases: the inflammatory phase, the proliferative phase and the maturation phase (Frangogiannis, 2014). The inflammatory phase is characterized by intense inflammation with the goal to clear all necrotic myocytes and debris. The proliferative phase is characterized by secretion of growth factors from monocytes-macrophages paired with vessel and myofibroblasts proliferation. Finally, the maturation phase is characterized by apoptosis of those cells and collagen turnover to form a mature scar. Gal-3 participates in all three phases and affects all the immune cells involved in the healing process (Meijers et al., 2015) (Figure 2).

Gal-3 levels increase after MI, both in cases of permanent coronary ligation (Sanchez-Mas et al., 2014; Li et al., 2023), and ischemia-reperfusion (Al-Salam and Hashmi, 2018; Mo et al., 2019; Zhang et al., 2020). In non-reperfused MI, Gal-3 peak was elevated at 24 h and peaked 1-week after MI in the infarct area, with a weaker and delayed increase observed in non-infarcted myocardium (Sanchez-Mas et al., 2014; Li et al., 2023). In the infarct area, the peak of Gal-3 was associated with an increase in markers of fibrosis and ECM remodeling such as transforming growth factor beta (TGF-β), fibroblast proliferation, collagen, fibronectin, alpha smooth muscle actin (αSMA), and metalloproteinase (Cassaglia et al., 2020; Frangogiannis, 2023; Wang et al., 2023). In the non-infarct area, Gal-3 increased expression was paired with infiltration of macrophages and tissue inhibitor of metalloproteinase-1 (TIMP-1), but not with the expression of collagens I and III (Sanchez-Mas et al., 2014). This divergence between Gal-3 expression in the infarct and non-infarct areas suggests a different involvement in the process of cardiac remodeling after MI. A study in 106 patients with chronic heart failure also established a direct relationship between plasma levels of Gal-3 and various molecular markers of ECM turnover, like the pro-collagen type III peptide (PIIINP), TIMP-1, and MMP-2 (Lin et al., 2009).

The dynamics of the inflammatory cellular infiltrate in the infarct area depend heavily on cytokines and chemokines. Neutrophils are among the first immune cells to infiltrate the necrotic myocardium and initiate cardiac healing. Although necessary, this early inflammation can also exacerbate tissue damage. Gal-3 can mediate neutrophil adhesion and recruitment to the injured myocardium. The expression of Gal-3, in addition with fibronectin and fibrinogen increase the expression in infiltrating neutrophils of the infarct zone and promote ECM reorganization (Daseke et al., 2019). It is accepted that neutrophils finish their role by the end of the first week after AMI, although recent data shows that these cells contribute to healing along with the temporal evolution of MI (Ma, 2021). Like macrophages, neutrophils can change its phenotype along the healing process, and contribute to the scar formation by switching from a pro-inflammatory to an anti-inflammatory profile (Daseke et al., 2021). However, the role of Gal-3 in that polarization is still unknown.

The second phase of post infarction wound healing is distinguished by the constitution of granulation tissue characterized by abundant macrophages, lymphocytes infiltration, neovascularization and fibroblast proliferation. The constitution of granulation tissue is a required step for the healing process. This stage is critical not only for the evolution of wound healing but also for its impact in the temporal evolution of global remodeling. From the second week of the temporal evolution of AMI, macrophages and lymphocytes create an inflammatory environment for neovascular formation, fibroblast proliferation and collagen synthesis by cytokine production. In this environment, newly formed blood vessels are necessary for supply to the healing zone with oxygen and nutrients (Prabhu and Frangogiannis, 2016). Increase in angiogenesis by SDS-1 at the beginning of AMI, improved the evolution of AMI and remodeling (Saxena et al., 2008). On the contrary, a reduction in neovascularization increased the infarct size and mortality (Tao et al., 2017). In this way, macrophages and lymphocytes begin the removal of necrotic myocytes; stimulate fibroblast proliferation, progressive collagen synthesis, and neovascularization. Macrophages and their precursors, monocytes, are integral parts of the innate immune response in cardiac reparative processes and have a direct influence on post-MI ventricular remodeling (Dick et al., 2019). While there is typically a small number of resident macrophages in the heart that participate in cardiac homeostasis, after myocardial infarction, many circulating monocytes infiltrate the infarcted myocardium, facilitated by chemotactic proteins like MCP-1/CCL2. These macrophages possess functional and phenotypic versatility that allows them to regulate the multiple processes that occur at each stage of repair and post-MI remodeling (Weinberger and Schulz, 2015). Macrophages play a key role in regulating inflammation by either promoting or inhibiting it. In the early stages of myocardial infarction, macrophages adopt a pro-inflammatory phenotype known as M1, which later transitions to an anti-inflammatory and profibrotic phenotype known as M2 (Kubota and Frangogiannis, 2022). Type 1 macrophages (M1) that govern the inflammatory stage secrete pro-inflammatory cytokines, chemokines, and growth factors to remove necrotic cells and degraded extracellular matrix. However, the persistence of these M1 macrophages over time or a significant decrease in their numbers could promote adverse ventricular remodeling. We showed that the decrease or excess of macrophage infiltration from the onset of myocardial infarction is critical to the evolution of ventricular remodeling (Gonzalez et al., 2005; Gonzalez et al., 2009). Studies from our group have demonstrated that Gal-3 promotes the recruitment of macrophages to the infarcted myocardium (Gonzalez et al., 2014), a critical event in the reparative phase following MI (Chen et al., 2022; Kubota and Frangogiannis, 2022). Gal-3 interacts with cell surface glycoconjugates, enabling adhesion and chemotaxis of macrophages to the injured tissue (Wang et al., 2023). Furthermore, Gal-3 influences macrophage polarization, skewing them towards a reparative M2 phenotype, which is associated with tissue remodeling and resolution of inflammation (Sun et al., 2023). Thus, Gal-3 emerges as a key regulator of macrophage-mediated wound healing processes in the context of post-infarction tissue repair. First, Gal-3 is involved in macrophage recruitment to the infarct area acting as a chemoattractant (Frangogiannis, 2014). Then, Gal-3 binds glycoconjugates on the surface of macrophages, facilitating their adhesion to the endothelium and subsequent migration into the injured area. This initial recruitment of macrophages is a crucial step in the inflammatory response and subsequent tissue repair. Gal-3 also influences the polarization of infiltrating macrophages by promoting the transition towards the M2 phenotype (Cassaglia et al., 2020). M2 macrophages are associated with anti-inflammatory and tissue-repairing functions, and plays a decisive role in resolving inflammation, clearing cellular debris, and facilitating tissue remodeling after MI. M2 macrophages are also involved in extracellular matrix deposition and fibrosis resolution, which are essential for restoring the structural integrity of the infarcted myocardium.

In summary, Gal-3 plays a multifaceted role in post-infarction wound healing by acting as both a chemotactic factor for macrophage recruitment and a modulator of their polarization toward the reparative M2 phenotype. This dual action of Gal-3 is pivotal in orchestrating the immune response and tissue repair processes following myocardial infarction, ultimately contributing to cardiac tissue recovery.

Studies from our group confirmed that Gal-3 is a critical regulator of wound healing and remodeling after AMI. Permanent coronary artery ligation is the most classical experimental model of AMI. This model is associated with large infarct, severely impaired cardiac function and adverse remodeling. As expected, Gal-3 KO mice showed reduced fibrosis in this model of AMI, but this reduction was associated with larger infarct, worse remodeling and a trend towards increased mortality. Gal-3 KO mice showed an altered dynamic of wound healing characterized by reduced macrophage infiltration, shifting to M2 macrophage phenotype, increased MMP-2 activity and reduced TGF-β (Cassaglia et al., 2020). On the contrary, Gal-3 selective cardiac overexpression leads to cardiac dysfunction *per se*, and these mice do not tolerate experimental AMI with an 80% mortality rate (Sonkawade et al., 2021). Finally, Gal-3 blockade with short harpin RNA (shRNA) was evaluated after AMI. shRNA against Gal-3 was associated with improved cardiac function, reduced remodeling, reduced macrophage infiltration and reduced fibrosis (Li et al., 2023). In summary, both complete absence and cardiac overexpression at the time of AMI are associated with deleterious effects after AMI with permanent coronary artery ligation, whereas inhibition with shRNA after AMI seems to be protective.

MI due to ischemia-reperfusion (I-R) is an experimental model that resembles clinical scenarios where patients are reperfused. This model is associated with a reduced scar and lesser cardiac dysfunction compared to permanent coronary artery ligation. Although a more translational model, some concern has raised due to cumulative failure to translate preclinical results to clinical practice (Jones et al., 2015; Lecour et al., 2021). Some studies have modulated Gal-3 in I-R. Pharmacologic Gal-3 inhibition with modified citrus pectin and G3-C12 showed reduction of infarct size and fibrosis, improved ventricular function, reduced fibrosis and reduced apoptosis (Ibarrola et al., 2019; Mo et al., 2019). Like pharmacologic inhibition, Gal-3 KO mice also showed reduced infarct size, inflammation and apoptosis in I-R (Zhang et al., 2020). One study -however- showed increased cardiac injury in Gal-3 KO mice associated with reduced inflammation and increased apoptosis (Al-Salam and Hashmi, 2018). Reduced inflammation is expected in Gal-3 KO mice, but it has been associated with reduced –not increased- injury in several models of I-R (Frangogiannis, 2007; Toldo et al., 2018; Andreadou et al., 2019). Besides, cardiac apoptosis can be non-cardiomyocyte specific and can be related to immune cells as part of the suppression of inflammation in Gal-3 KO mice. Overall, these results should be interpreted with caution. The discrepancy in Gal-3 KO mice between reperfused and non-reperfused MI can be partially explained by the role of fibrosis and the scar in each model; while the scar is transmural in non-reperfused large MI and needs to provide adequate tension, a patchy and subendocardial scar is observed in reperfused MI (Lindsey et al., 2021). Thus, the risk of ventricular rupture, aneurysm and dyskinesia are only present in non-reperfused models.

Overall, the results of Gal-3 modulation in AMI reinforce the need for a balanced inflammatory response after AMI and underline the complexity of Gal-3. Due to its non-carbohydrate binding properties, genetic overexpression or deletion leads to different results than pharmacological exogenous blockade. While pharmacologic inhibition seems more translational, the specificity of blockers to selectively target Gal-3 has been challenged (Stegmayr et al., 2016; Kirk and de Boer, 2019). On the other hand, absence of Gal-3 in KO mice is a model of complete blockade, but mice are born with the alteration before AMI, leading to compensatory mechanisms or inability to form any mature scar. The hybrid approach of genetic blockade with rhRNA deserves further attention: it can be started after AMI and it blocks both carbohydrate-dependent and independent functions. However, suppression of Gal-3 is not complete compared to KO mice. Finally, it is important to determine the timing for Gal-3 modulation and blockade. It seems that some degree of Gal-3 activation is necessary before/at the time of AMI in non-reperfused AMI only, whereas overexpression in the same model is detrimental. RNA inhibition after AMI is protective in this model, while pharmacologic inhibition is also protective but has only been tested in I-R.

### 3.2 Hypertension

The first association between Gal-3 and cardiovascular disease was reported in 2004 in homozygous transgenic TGRmRen2-27 (Ren-2) rats. This experimental model is characterized by severe hypertension that is first associated with cardiac hypertrophy, followed by cardiac dilation and dysfunction (Langheinrich et al., 1996). Interestingly, while some rats developed dyspnea and hemodynamic compromise after 15 weeks, others remained compensated. Both groups of animals were compared using a total DNA array of cardiac tissue, observing that 48 genes were differentially expressed. These genes encode various ECM proteins including different types of collagen, osteoactivin, and fibronectin. Gal-3 was the most overexpressed gene in decompensated hearts, with values five times higher than controls (Sharma et al., 2004). In this same model, pharmacologic blockade of Gal-3 with N-acetyllactosamine led to improved ventricular function and reduced myocardial collagen content (Yu et al., 2013; Frenay et al., 2015). These results were also observed in another similar model of severe hypertension and hypertrophy followed by heart failure, which is Dahl rats plus high sodium diet (Klotz et al., 2006). In this model, blockade of Gal-3 with *in vivo* injections of shRNA led to improved survival and reduced markers of cardiomyocyte apoptosis (Li et al., 2018). Of note, no evaluation of cardiac function and geometry was performed in this study.

Aldosterone infusion and salt diet are associated with hypertension, concentric hypertrophy cardiac inflammation and fibrosis but without severe cardiac dysfunction (Lopez-Andres et al., 2011). Moreover, aldosterone infusion alone leads to cardiac inflammation and fibrosis in the absence of hypertension. In this later model, Gal-3 KO mice were protected from all these effects on cardiac fibrosis and inflammation (Calvier et al., 2015; Martinez-Martinez et al., 2015). Pharmacologic blockade of Gal-3 with modified citrus pectin (MCP) also reduced inflammation and fibrosis both in this model and in genetic models of hypertension like spontaneous hypertensive rats (SHR). Finally, the effects of Gal-3 were also studied in other models of hypertension related to the renin-angiotensin system. After chronic infusion of angiotensin II (ANG II), Gal-3 KO mice showed reduced infiltration cardiac macrophages and inflammation, leading to improved cardiac function and reduced fibrosis, albeit no effect on cardiac hypertrophy and blood pressure was observed (Gonzalez et al., 2016). Macrophage infiltration are critical in the development of target organ damage (Sharma et al., 2008). Gal-3 increased the migration of macrophages and TNF-α release both *in vivo* and *in vitro* (Sharma et al., 2008). Previous studies from our group showed that lack of Gal-3 prevented the myocardial macrophages infiltration, MCP-1 expression and dysfunction. Targeted inhibition of Gal-3 attenuated macrophages infiltration, IL-6, MCP-1 levels, renal structural and functional damage in Ren-2 rats with hypertensive end-organ damage (Frenay et al., 2015). However, the exact mechanisms through which Gal-3 influences macrophages in hypertension are not fully understood and may vary in different contexts. It is possible that Gal-3 contributes to the activation of inflammatory pathways, leading to macrophage recruitment and polarization towards a pro-inflammatory state (M1).

The effects of Gal-3 modulation on blood pressure are more controversial: MCP reduced blood pressure in aldosterone and salt diet (Calvier et al., 2015) but did not modify blood pressure in SHR (Martinez-Martinez et al., 2015), and absence of Gal-3 in KO mice had no effect after ANG II infusion (Gonzalez et al., 2016). These differences are probably related to the experimental model used and the degree of hypertension (Lerman et al., 2019).

The anti-inflammatory and anti-fibrotic effects of Gal-3 inhibition are not cardiac specific, but likely systemic. These include, among others, improved endothelium function, reduced fibrosis and oxidative stress in blood vessels (Calvier et al., 2013; Pang et al., 2023) and reduced kidney injury (Calvier et al., 2015; Frenay et al., 2015; Martinez-Martinez et al., 2018). In summary, there is concordant experimental evidence showing a beneficial effect of Gal-3 inhibition to prevent cardiac damage after hypertension, while the results on blood pressure are more controversial.

### 3.3 Atherosclerosis

Atherosclerosis is a complex and multifactorial disease characterized by the generation of plaque in arterial walls, leading to a progressive narrowing and stiffness of the arteries. Inflammation and immune responses play crucial roles in the development and progression of atherosclerosis. Thus, atherosclerosis is a chronic and silent process that ultimately leads to atherosclerotic plaque rupture and vessel thrombosis (Soehnlein and Libby, 2021), causing both acute myocardial infarction and ischemic stroke, which are the leading causes of mortality in the modern world (Hansson, 2005). Inflammation orchestrates both development of atheroma and plaque rupture (Libby, 2021). The association between Gal-3 and atherosclerosis has been widely studied (Gao et al., 2020). The involvement of Galectin-3 in atherosclerosis is thought to be linked to its pro-inflammatory and pro-fibrotic properties. Gal-3 levels are increased in atheromas of mice and it is more concentrated in unstable regions of human carotid atheromas (Papaspyridonos et al., 2008). Galectin-3 has been associated with various aspects of atherosclerotic plaque formation and stability by regulates endothelial dysfunction, inflammation, oxidative stress, lipid endocytosis, and VSMC migration. Gal-3 promote the adhesion between neutrophils and endothelial cells thought integrin in the cell surface modifying cell-cell interaction (Sato et al., 2002; Nachtigal et al., 1998), Gal-3 is mainly present in macrophages and foam cells of the atherosclerotic plaques and such increase was positively correlated with the severity of the plaque. The differentiation of macrophages into foam cells by increasing the lipoprotein intake was promoted by Gal-3 (Zhu et al., 2001). Exogenous Gal-3 promoted the proliferation of vascular small muscle cells, which are particularly important cells in plaque stabilization (Tian et al., 2017). However, conflicting results were also observed in Gal-3 KO mice with different experimental models of atherosclerosis. One study showed that Gal-3 KO mice fed with a high fat diet showed more complex ‘lesions’ in the aortic sinuses compared to WT (Iacobini et al., 2009). However, diet alone is usually not an established model of atherosclerosis and may represent the beginning of the disease. Most used models combined ApoE knockout mice with fat diet. Double knockout mice (ApoE and Gal-3) with standard diet did not develop atherosclerosis in the long term, as opposed to ApoE knockout expressing Gal-3 (Nachtigal et al., 2008). Finally, the same double ApoE model but fed with a high-cholesterol “western” diet showed reduced atherosclerosis in the absence of Gal-3 (MacKinnon et al., 2013). However, a more recent study in the same model showed that double knockout mice led to more unstable atheroma’s with larger necrotic core and less fibrosis (Panidis et al., 1988). Finally, pharmacologic inhibition of Gal-3 with MCP also led to reduced atherosclerosis in two studies in ApoE mice with fat diet (MacKinnon et al., 2013; Lu et al., 2017).

### 3.4 Other cardiomyopathies

Gal-3 modulation alters cardiac remodeling and function in different models of experimental cardiomyopathy. Isoproterenol (Iso) is an adrenergic drug, and repeated Iso injections are a validated model of cardiomyopathy associated with focal areas of myocardial necrosis, inflammation and fibrosis (Balakumar et al., 2007). Iso induces cardiac inflammation with NLRP3 inflammasome activation followed by IL-18, finally leading to Gal-3 expression (Hu et al., 2023). However, Gal-3 is also increased after Iso by several mechanisms (Vergaro et al., 2016; Nguyen et al., 2018) including the β‐adrenoceptor‐Mst1(Hippo)‐YAP pathway (Zhao et al., 2019). Gal-3 KO mice showed reduced dysfunction and inflammation after isoprenaline, another β-adrenergic (Zhao et al., 2019), which was confirmed in several models of Gal-3 blockade with MCP (Vergaro et al., 2016; Xu et al., 2020; Li et al., 2021).

Streptozotocin (STZ) is a drug that causes pancreatic b cells necrosis, thus mimicking type 1 diabetes and diabetic cardiomyopathy (DCMP). Gal-3 KO mice showed less hyperglycemia and less pancreatic injury after STZ due to reduced inflammation (Mensah-Brown et al., 2009), but Gal-3 overexpression in pancreatic b cells led to reduced apoptosis and resistance to STZ damage (Jovicic et al., 2021). Regarding DCMP and Gal-3, results are more consistent: Gal-3 KO mice had less myocardial injury, better remodeling and cardiac function, and less interstitial fibrosis (Zhu et al., 2022). This protective effect was mediated by reduced oxidative stress and apoptosis. Pharmacologic Inhibition with MCP showed similar results (Sun et al., 2020).

Trypanozoma Cruzi (T. Cruzi) is the parasite responsible for Chagas’ disease and cardiomyopathy. The disease appears several years after the infection, and it is mediated by immune activation rather than direct parasite myocardial damage (Bern, 2015). Gal-3 is expressed in areas of inflammation of hearts from patients with terminal Chagas’ disease (Souza et al., 2017a). Moreover, Gal-3 promotes T. Cruzi infection (Poncini et al., 2021). Pharmacologic Gal-3 inhibition with N-acetyl-b-Lactosamine was associated with reduced oxidative stress and fibrosis in chronic infection, although no improvements were observed in physical capacity and arrhythmias (Souza et al., 2017a). Gal-3 KO mice showed increased survival and reduced parasite load after T. Cruzi infection (Chain et al., 2020). However, Gal-3 is important for the immunomodulatory effects of mesenchymal stromal cells (MSC). Knockout of Gal-3 in these cells impaired their capacity to suppress inflammation in chronic Chagas disease, although *in vivo* T Cruzi survival was also impaired in these MSC (Souza et al., 2017b).

Doxorubicin (Dox) is an antiproliferative drug commonly used for some blood and breast cancers. Although effective, Dox has important cardiac toxicity that limits its use and Dox injection is an established experimental model of cardiomyopathy (Chatterjee et al., 2010). The mechanisms responsible for cardiac injury are not fully understood, but include oxidative stress, direct DNA damage, inflammation, apoptosis and fibrosis. There are two models of dox-induced cardiomyopathy: acute injury (single high dose injection) and chronic administration. As mentioned above, Gal-3 participates both in inflammation and fibrosis. Data on Gal-3 KO mice after single Dox injection is controversial. One study showed reduced inflammation, which is expected due to Gal-3’s role in promoting inflammation, but paired with increased mortality, oxidative stress and cardiac damage (Al-Salam et al., 2022). On the contrary, our group observed reduced oxidative stress and cardiac damage that led to improved cardiac function in Gal-3 KO mice (Seropian et al., 2023). Although both studies found reduced expression of cardiac inflammation in the study of Al-Salam, Gal-3 KO mice treated with Dox showed increased mortality, cardiac damage and oxidative stress. Thus, it is difficult to understand such divergence because it is widely accepted that Gal-3 promotes oxidative stress in immune cells and several studies have shown that reducing the inflammation and oxidative stress after Dox leads to better outcomes (Zhang et al., 2011; Zhang et al., 2021).

Our results are in line with a study that treated rats with MCP and observed reduced oxidative stress and improved cardiac function (Tian et al., 2020).

Several experimental models of myocarditis exist. Gal-3 is upregulated in areas of inflammation in the heart after experimental encephalomyocarditys, and those Gal-3 positive areas are later turn into fibrosis (Noguchi et al., 2019), which is associated with increased macrophage infiltration (Jaquenod De Giusti et al., 2015). Gal-3 KO mice showed reduced inflammation and fibrosis in myocarditis induced by coxsackievirus (Jaquenod De Giusti et al., 2015). However, Gal-3 KO mice showed increased cardiac inflammation in autoimmune experimental myocarditis. Importantly, inflammation was shifted towards a resolutive phenotype including M2 macrophages and Th-2 cytokines (Kovacevic et al., 2018). These differences may be related to the model of myocarditis used (viral vs. autoimmune).

Finally, cardiac overexpression of Gal-3 *per se* leads to cardiomyopathy characterized by hypertrophy and reduced contractility (Sonkawade et al., 2021). As mentioned above (see hypertension section), Gal-3 infusion in the pericardium leads to cardiac dysfunction and fibrosis *per se* (Sharma et al., 2004; Liu et al., 2009), thus becoming an experimental model of cardiomyopathy.

## 4 Galectin-3 in cardiac aging

The natural evolution of aging significantly affects the cardiovascular system, increasing the risk of developing cardiac pathologies and heart failure. Age is one of the most important determinants of cardiovascular health and an important cardiovascular risk factor. For this reason, the study of the underlying mechanisms associated with cardiac aging has relevant clinical implications (Dao Fu et al., 2012; Abdulla et al., 2020). Constitutive aging of the heart increases its susceptibility to stress and contributes to increased cardiovascular morbidity and mortality in the elderly (Abdulla et al., 2020). A hallmark of cardiac aging is progressive ventricular remodeling characterized by myocardial hypertrophy, interstitial fibrosis and lately ventricular dysfunction (Loffredo et al., 2014). The underlying mechanisms are complex and involve inflammation, loss of cardiomyocytes by apoptosis and alterations in autophagy (Akihiro et al., 2016). The intracellular pathways involved in cardiomyocyte loss is not fully understood. ANG II, the main effector peptide of the renin RAS, is a major hormone contributing to myocardial hypertrophy and fibrosis, and it is strongly implicated in aging-related ventricular remodeling (Dao Fu et al., 2012; Abdulla et al., 2020). Chronic pharmacological inhibition of the RAS prevented adverse cardiac remodeling associated with aging and significantly improved survival (Nidia et al., 2005). As shown above (see hypertension section) modulation of the RAS are a valid model of hypertension, and Gal-3 inhibition has shown protective results in these models as well (Gonzalez et al., 2016).

Recent studies showed that the absence of Gal-3 in aged mice exacerbates kidney fibrosis, oxidative stress, and promotes renal dysfunction, suggesting that Gal-3 may be protective for kidney aging (Iacobini et al., 2005). We recently studied the cardiovascular phenotype of aged Gal-3 KO mice (Fontana Estevez et al., 2022). At 2 years old, Gal-3 KO mice showed a significantly decreased in survival. Moreover, Gal-3 KO mice showed exacerbated cardiac hypertrophy and interstitial fibrosis. Besides, cardiac apoptosis was increased in Gal-3 KO mice, while sirtuins were decreased. Interestingly, absence of Gal-3 led to increased myocardial expression of ANG II, but no effects were observed in blood pressure. This suggests that Gal-3 plays a pivotal role in the temporal evolution of cardiac aging. The likely regulatory link between Gal-3 and ANG II is of paramount relevance in cardiac aging but the mechanism is still unknown. Importantly, the role of Gal-3 depends on the conditions where it is being studied. The same Gal-3 KO model has shown very different phenotypes depending on the experimental model tested, as demonstrated along this review.

## 5 Concluding remarks

Gal-3 is associated with inflammation, fibrosis, remodeling and dysfunction in cardiovascular pathophysiology. Gal-3 actives all cardiac cells and exhibits a remarkable plasticity of action in the temporal evolution of cardiac remodeling. The relevant participation of Gal-3 in the pathophysiology of cardiac remodeling underlines the importance of deeply understanding the cardiac role of this lectin. Unraveling the mechanism of action of Gal-3 in the heart highlights its potential therapeutic benefit to reduce heart failure.
